# Dopexamine and norepinephrine versus epinephrine on gastric perfusion in patients with septic shock: a randomized study [NCT00134212]

**DOI:** 10.1186/cc4827

**Published:** 2006-02-16

**Authors:** Philippe Seguin, Bruno Laviolle, Patrick Guinet, Isabelle Morel, Yannick Mallédant, Eric Bellissant

**Affiliations:** 1 Service de Réanimation Chirurgicale INSERM U620, Hôpital de Pontchaillou, Université de Rennes 1, Rennes, France; 2 Centre d'Investigation Clinique INSERM 0203, Unité de Pharmacologie Clinique, Hôpital de Pontchaillou, Université de Rennes 1, Rennes, France; 3 Laboratoire des Urgences & Réanimations, Hôpital de Pontchaillou, Université de Rennes 1, Rennes, France

## Abstract

**Introduction:**

Microcirculatory blood flow, and notably gut perfusion, is important in the development of multiple organ failure in septic shock. We compared the effects of dopexamine and norepinephrine (noradrenaline) with those of epinephrine (adrenaline) on gastric mucosal blood flow (GMBF) in patients with septic shock. The effects of these drugs on oxidative stress were also assessed.

**Methods:**

This was a prospective randomized study performed in a surgical intensive care unit among adults fulfilling usual criteria for septic shock. Systemic and pulmonary hemodynamics, GMBF (laser-Doppler) and malondialdehyde were assessed just before catecholamine infusion (T_0_), as soon as mean arterial pressure (MAP) reached 70 to 80 mmHg (T_1_), and 2 hours (T_2_) and 6 hours (T_3_) after T_1_. Drugs were titrated from 0.2 μg kg^-1 ^min^-1 ^with 0.2 μg kg^-1 ^min^-1 ^increments every 3 minutes for epinephrine and norepinephrine, and from 0.5 μg kg^-1 ^min^-1 ^with 0.5 μg kg^-1 ^min^-1 ^increments every 3 minutes for dopexamine.

**Results:**

Twenty-two patients were included (10 receiving epinephrine, 12 receiving dopexamine–norepinephrine). There was no significant difference between groups on MAP at T_0_, T_1_, T_2_, and T_3_. Heart rate and cardiac output increased significantly more with epinephrine than with dopexamine–norepinephrine, whereas. GMBF increased significantly more with dopexamine–norepinephrine than with epinephrine between T_1 _and T_3 _(median values 106, 137, 133, and 165 versus 76, 91, 90, and 125 units of relative flux at T_0_, T_1_, T_2 _and T_3_, respectively). Malondialdehyde similarly increased in both groups between T_1 _and T_3_.

**Conclusion:**

In septic shock, at doses that induced the same effect on MAP, dopexamine–norepinephrine enhanced GMBF more than epinephrine did. No difference was observed on oxidative stress.

## Introduction

In septic shock, when volume resuscitation fails to restore mean arterial pressure (MAP), catecholamines such as dopamine, dobutamine, epinephrine (adrenaline), or norepinephrine (noradrenaline) are used, either alone or in combination [1-3]. Their effectiveness primarily reflects their cardiac and vascular actions, but their ability to modulate the sepsis-induced production of reactive oxygen species may also participate [4]. Nonetheless, if they generally allow normalizing MAP, they can leave some regional blood flows impaired, especially hepatosplanchnic perfusion, which contributes to multiple organ failure [5,6].

Dopexamine is a structural and synthetic analog of dopamine that exerts systemic vasodilatation through the stimulation of β_2 _adrenoceptors and peripheral DA_1 _and DA_2 _receptors, and weak inotropic properties through the stimulation of β_1 _adrenoceptors. This pharmacological profile could make the use of dopexamine interesting in combination with norepinephrine to improve both systemic hemodynamics and microcirculatory blood flow, notably gut perfusion. Moreover, in rats, dopexamine has been shown to exert a protective effect against the reactive oxygen species generated by an intravenous administration of xanthine followed by xanthine oxidase [7]. The main objective of the present study was therefore to compare the effects of the combination of dopexamine and norepinephrine with those of epinephrine alone on gastric mucosal blood flow (GMBF). The effects of these drugs on oxidative stress were also assessed.

## Materials and methods

### Protocol and approval

The protocol was approved by the institutional review board for human research of our hospital (Comité Consultatif de Protection des Personnes dans la Recherche Biomédicale de Rennes) on September 5^th ^2001 (Reference number: 01/34-355). The study was prospective, randomized, and open-labeled, and was performed on two parallel groups. It was conducted in a 21-bed surgical intensive care unit in a university hospital. Informed consent was obtained from each patient or next of kin.

### Patients

#### Inclusion criteria

Adults aged over 18 years were included if they fulfilled the following:

(1) Evidence of infection.

(2) At least three of the following criteria: temperature more than 38.0°C or less than 36.5°C, respiratory rate more than 20 breaths per minute or arterial pressure in CO_2 _(PaCO_2_) less than 32 mmHg or mechanical ventilation, heart rate more than 90 beats per minute, white blood cell count more than 12,000 per mm^3 ^or less than 4,000 per mm^3^.

(3) At least two of the following criteria: plasma lactate more than 2 mmol per liter or unexplained metabolic acidosis (pH < 7.3), hypoxemia defined by arterial pressure in oxygen (PaO_2_) less than 70 mmHg at room air or a ratio of PaO_2 _to fractional inspired oxygen (FiO_2_) of less than 280 mmHg (or less than 200 mmHg if pneumonia was the source of sepsis) or a need for mechanical ventilation, urine output less than 30 ml per hour for at least 2 hours despite a fluid challenge of at least 500 ml, a platelet count of less than 100,000 per mm^3 ^or a decrease of 50% from a previous value or unexplained coagulopathy (prothrombin time less than 60% and elevated fibrin degradation products more than 10 μg per ml).

(4) Systolic blood pressure less than 90 mmHg despite an optimal volume loading defined by a pulmonary capillary wedge pressure more than 14 mmHg.

#### Non-inclusion criteria

Pregnant women and patients who had a history of esophageal or gastric disease were not included; neither were patients with a history of esophageal or gastric surgery.

### Data collection at inclusion

The following data were recorded at inclusion: general characteristics (age, weight, height, and sex); severity of illness assessed by vital signs, Simplified Acute Physiology Score II (SAPS II), and Sequential Organ Failure Assessment (SOFA) score; and interventions including administered drugs, volume of fluid infusion during the previous 24 hours, and mechanical ventilation conditions. Moreover, blood samples were drawn for hematological and biochemical analyses and blood cultures, and specimens from the site of infection were collected systematically.

### Investigated variables

#### Systemic and pulmonary hemodynamics

All patients had an arterial catheter (Seldicath 4F 3874 13; Plastimed Laboratories, Saint-Leu-La-Forêt, France) and a pulmonary arterial catheter (ref. 831F35; Baxter Healthcare Corporation, Irvine, CA, USA) connected to a monitor (7000/SC 9000XL; Siemens-Elema AB, Solna, Sweden) allowing measurements of heart rate and arterial pressures (systolic and diastolic systemic arterial pressures, right atrial pressure, systolic and diastolic pulmonary arterial pressures, and pulmonary capillary wedge pressure). Calibration was performed with reference to the mild axillary line. Pulmonary capillary wedge pressure was measured at the end of expiration. Cardiac output was measured by thermodilution in triplicate with 10 ml of ice-cold (less than 5.0°C) 5% dextrose solution injected asynchronously with the respiratory cycle. MAP and mean pulmonary arterial pressure, stroke volume, and systemic and pulmonary vascular resistances were calculated from standard formulae.

#### Gastric mucosal blood flow

GMBF was assessed with a laser-Doppler flowmeter (Periflux PF3; Perimed, Stockholm, Sweden) as described previously [8,9]. In brief, the laser light is conducted and transmitted to the tissue by an optic fiber (probe 324). Two signals are available for external recording. One signal is proportional to the number and velocity of the red blood cells moving in the measured volume (about 1 mm^3^) and the other allows the determination of whether the optical probe is making adequate contact with tissue surface. The flow value is expressed in units of relative flux (perfusion units). Calibration was performed against a standard latex solution before the start of measurements, as recommended by the manufacturer. Then the laser-Doppler probe was pushed through the noose into the stomach, the position being checked with X-rays. All patients had simultaneously a nasogastric tube suctioning at -60 mmHg. The laser-Doppler signal was recorded on a computer. Special care was taken to ensure persistent contact between the probe and the gastric mucosa. The ratio between GMBF and cardiac output was calculated.

#### Blood gases and arterial lactate

Arterial and mixed venous blood gases were determined from samples collected anaerobically in heparinized plastic syringes through the arterial and distal port of the pulmonary artery catheter, respectively. Samples were analyzed within 15 minutes by a co-oximeter (Abl 725; Radiometer, Copenhagen, Denmark) to determine arterial and mixed venous oxygen tension and saturation, as well as arterial lactate concentration (enzymatic dosage). Arterial and mixed venous oxygen content, oxygen delivery, and oxygen consumption were calculated from standard formulae.

#### Oxidative stress

Oxidative stress was assessed from plasma malondialdehyde levels, as an index of lipid peroxidation induced by the generation of free radicals. Malondialdehyde concentrations were estimated by a colorimetric test with thiobarbiturate [10]. After precipitation of protein with a mixture of phosphotungstic acid and sulfuric acid, the supernatant was incubated with thiobarbiturate for one hour at 90°C. Thiobarbiturate-reactive substances were then extracted with n-butanol and the absorbance was monitored at 535 nm. The concentrations were calculated from a calibration curve obtained by the acid hydrolysis of 1,1,3,3-tetramethoxypropane solution, generating standard concentrations of malondialdehyde. Standards were then treated with thiobarbiturate reagent and extracted with n-butanol in the same way as unknown samples. The normal value of malondialdehyde in healthy subjects was less than 4 μmol per liter.

### Experimental protocol and treatments

**Figure 1:**
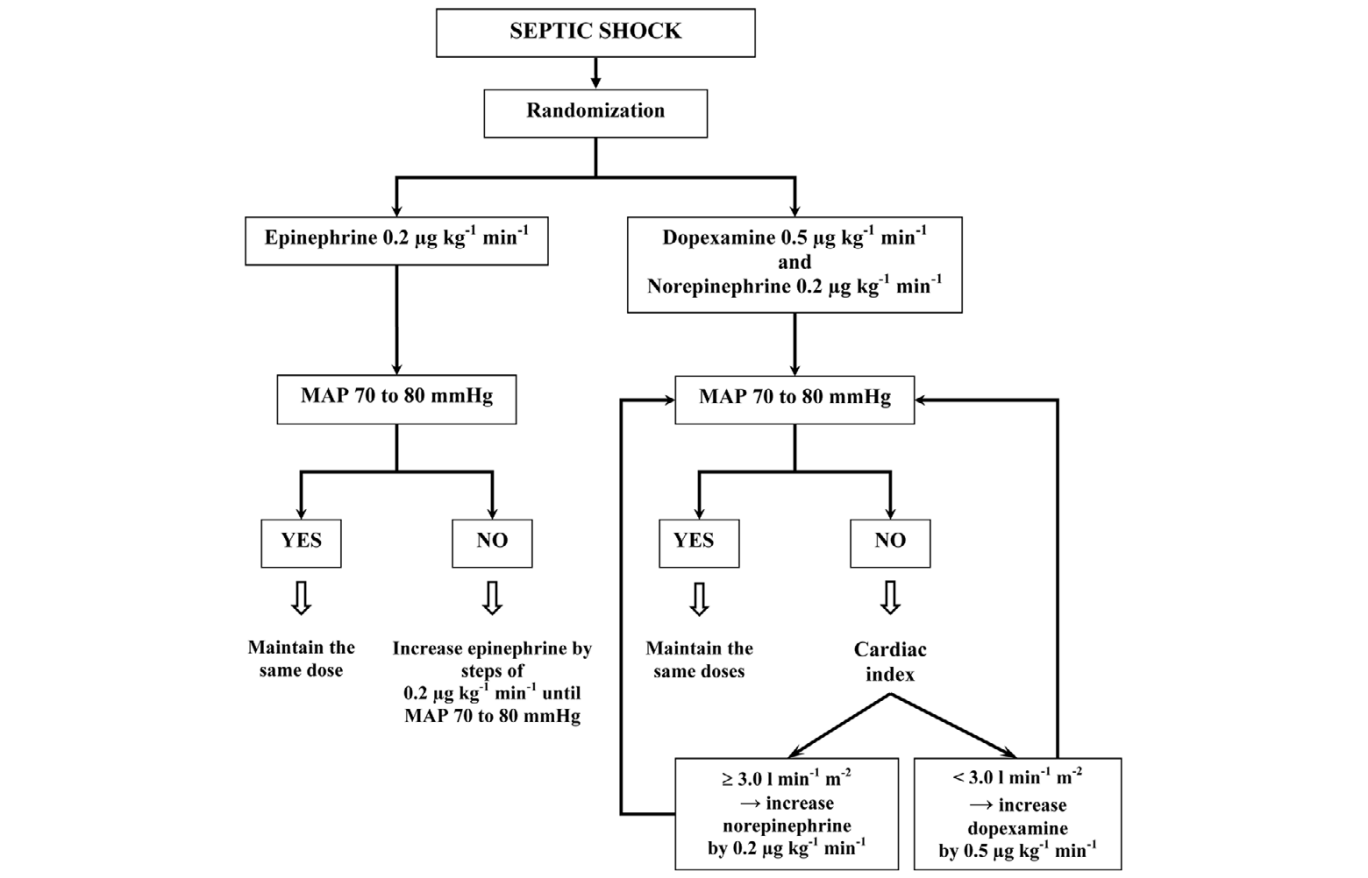
Algorithm of doses adaptation in the two groups. MAP, mean arterial pressure.

As soon as inclusion criteria had been checked and informed consent had been obtained, baseline measurements were performed, including systemic and pulmonary hemodynamics and GMBF, and blood samples were drawn (T_0_). Ventilator settings were adapted for each patient so as to reach arterial oxygen saturation above 90% and a plateau pressure lower than 30 cmH_2_O. Patients were then randomized to receive either epinephrine alone or a combination of dopexamine and norepinephrine. The unpredictability of randomization was guaranteed by two specific procedures: the randomization list, generated with a computer, was equilibrated using unequal-sized blocks and the randomization of a patient was performed by an independent pharmacist. Study treatments were administered with an automatic syringe through the intermediate port of the pulmonary artery catheter. Drugs were titrated from 0.5 μg kg^-1 ^min^-1 ^with 0.5 μg kg^-1 ^min^-1 ^increments every 3 minutes for dopexamine and from 0.2 μg kg^-1 ^min^-1 ^with 0.2 μg kg^-1 ^min^-1 ^increments every 3 minutes for norepinephrine and epinephrine, until MAP reached 70 to 80 mmHg. If necessary, dopexamine and norepinephrine doses were adjusted by using cardiac output according to the algorithm in Figure 1. When MAP was above 80 mmHg, the dose of norepinephrine or epinephrine was adjusted to let MAP decrease to between 70 and 80 mmHg. Once the target MAP had been obtained, the treatment was maintained at the same doses, and the same measurements as at baseline were performed (T_1_). No adjustment of fluid infusion or mechanical ventilation was allowed during this first study period (namely between T_0 _and T_1_). The same variables were measured two hours (T_2_) and six hours (T_3_) later. During this second study period (that is, between T_1 _and T_3_), fluid loading and doses of catecholamines were adjusted to maintain a pulmonary capillary wedge pressure of more than 14 mmHg and MAP between 70 and 80 mmHg, respectively.

### Sample size

Sample size estimation was based on GMBF data from our previous study, in which the standard deviation of GMBF was 160 units at inclusion [9]. In the actual protocol, 20 patients were planned to be included so as to detect a difference between the two groups of 240 units with a type I error of 5% and a power of 95%.

### Statistical analysis

Statistical analysis was performed with SAS statistical software V8.02 (SAS Institute, Cary, NC, USA). Data are presented as means ± SD for normally distributed variables and as medians (25th – 75th centiles) for non-normally distributed variables. The homogeneity of pretreatment (T_0_) mean values between groups was tested with Student's *t *test or Wilcoxon's rank sum test when needed. Comparisons of treatment mean values between groups over the second study period (that is, between T_1 _and T_3_) were performed with a two-way (time, treatment) analysis of covariance (mixed model), the analysis being adjusted on baseline values. In case of a significant time–treatment interaction, treatment effect was assessed time by time by a one-way analysis of covariance similarly adjusted on baseline values. For non-normally distributed variables a non-parametric repeated-measures analysis, also adjusted on baseline values (mixed model), was performed on ranked data. For all analyses, *p *< 0.05 was considered to be significant.

## Results

A total of 22 patients were randomized (10 received epinephrine, and 12 received dopexamine–norepinephrine) between March 25^th ^2002 and March 17^th ^2004. Two patients (one in each group) were excluded from the analysis on the main endpoint because of inadequate location of the laser-Doppler probe, and two patients (one in each group) could not be investigated at T_3 _because of the need for prompt surgical management to control the source of sepsis.

### General characteristics of study patients at inclusion

There was no significant difference between the groups in age, weight, height, sex ratio, SAPS II, SOFA, volume of fluid infusion during the previous 24 hours, and mechanical ventilation conditions (Table 1). The origin of sepsis was essentially peritonitis (six patients in the epinephrine group and nine patients in the dopexamine–norepinephrine group). The infection was not microbiologically documented in one patient in the epinephrine group and in two patients in the dopexamine–norepinephrine group. Mortality rates at days 28 and 90 were 3/10 (30%) and 4/10 (40%), respectively, in the epinephrine group, and 2/12 (17%) and 3/12 (25%), respectively, in the dopexamine–norepinephrine group.

The median (25th – 75th centiles) delay between randomization and stabilization of MAP at the target level was 60 minutes (50 – 80 minutes) and 70 minutes (60 – 140 minutes) in the epinephrine and dopexamine–norepinephrine groups, respectively (*p *= 0.078). Median catecholamine doses (μg kg^-1 ^min^-1^) at the three times of investigation were 0.17 (0.14 to 0.19) at T_1_, 0.19 (0.14 to 0.24) at T_2 _and 0.19 (0.18 to 0.21) at T_3 _for epinephrine, 0.51 (0.48 to 0.53) at T_1_, 0.51 (0.49 to 0.53) at T_2 _and 0.51 (0.50 to 0.55) at T_3 _for dopexamine, and 0.20 (0.11 to 0.60) at T_1_, 0.20 (0.12 to 0.69) at T_2 _and 0.18 (0.11 to 0.74) at T_3 _for norepinephrine.

### Effects of treatments on systemic and pulmonary hemodynamics and oxygenation parameters

At baseline, there was no significant difference between the two groups whichever variable was considered (Table 2). There was also no significant difference in MAP between the two groups at T_1_, T_2_, and T_3_. Heart rate and cardiac output increased significantly more with epinephrine than with dopexamine–norepinephrine between T_1 _and T_3 _(+5%, *p *= 0.023 for treatment effect, and +13%, *p *= 0.039, respectively). Similarly, oxygen delivery and oxygen consumption increased significantly more with epinephrine than with dopexamine–norepinephrine between T_1 _and T_3 _(+17%, *p *= 0.009 for treatment effect, and +34%, *p *= 0.001, respectively).

### Effects of treatments on GMBF and on the ratio between GMBF and cardiac output

**Figure 2:**
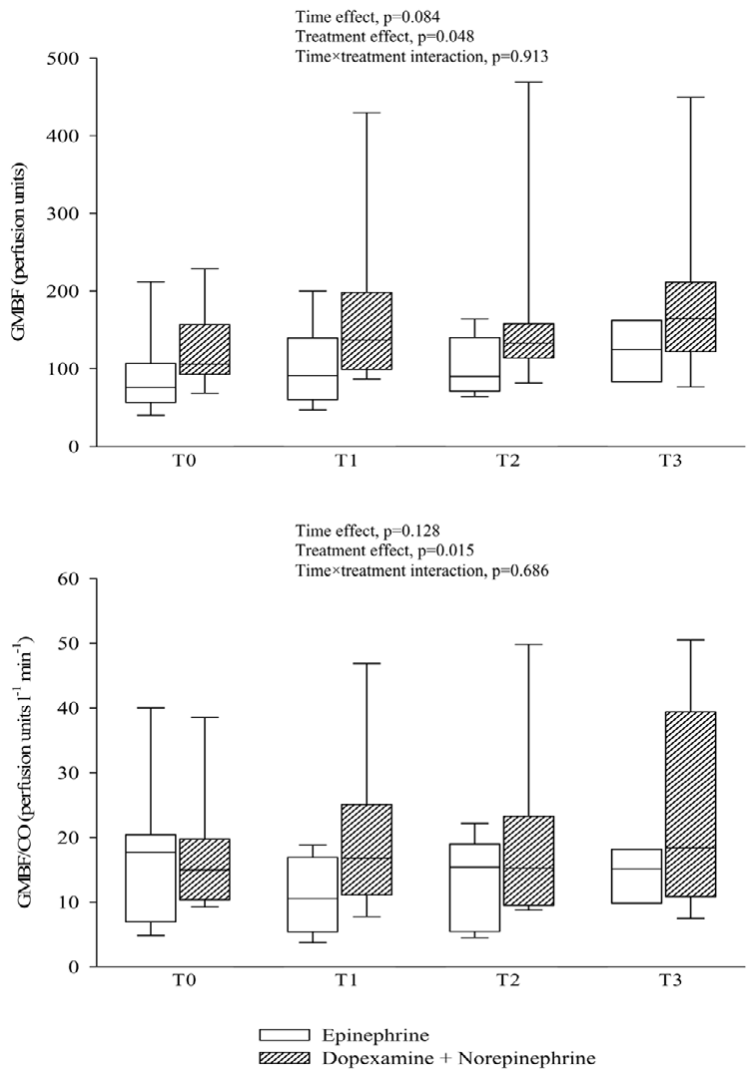
Evolution of gastric mucosal blood flow (GMBF) and ratio between GMBF and cardiac output. T_0_, just before catecholamine infusion; T_1_, as soon as mean arterial pressure reached 70 to 80 mmHg; T_2_, 2 hours after T_1_; T_3_, 6 hours after T_1_. Data are presented as boxplots. CO, cardiac output.

At baseline there was no significant difference in GMBF or in the ratio between GMBF and cardiac output between the two groups (Table 2). GMBF increased significantly more with dopexamine–norepinephrine than with epinephrine (medians 106, 137, 133, and 165 compared with 76, 91, 90, and 125 units of relative flux at T_0_, T_1_, T_2_, and T_3_, respectively; *p *= 0.048 for treatment effect; Figure 2, top). The ratio between GMBF and cardiac output decreased with epinephrine, whereas it did not change with dopexamine–norepinephrine between T_1 _and T_3 _(*p *= 0.015 for treatment effect; Figure 2, bottom).

### Effects of treatments on arterial lactate concentration and oxidative stress

**Figure 3:**
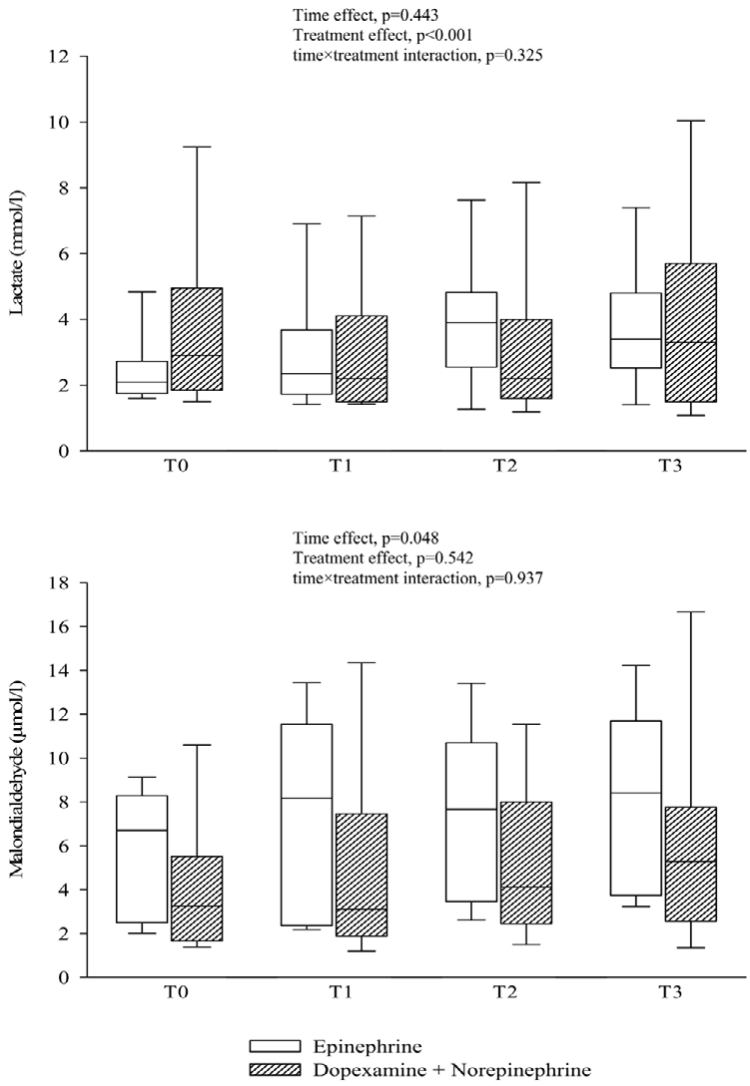
Evolution of arterial lactate and malondialdehyde concentrations. T_0_, just before catecholamine infusion; T_1_, as soon as mean arterial pressure reached 70 to 80 mmHg; T_2_, 2 hours after T_1_; T_3_, 6 hours after T_1_. Data are presented as boxplots.

At baseline, there was no significant difference in arterial lactate and malondialdehyde concentrations between the two groups. Arterial lactate increased with epinephrine, whereas it did not change with dopexamine–norepinephrine between T_1 _and T_3 _(*p *< 0.001 for treatment effect; Figure 3, top). Malondialdehyde similarly increased in the two groups between T_1 _and T_3 _(*p *= 0.048 for time effect; *p *= 0.542 for treatment effect; Figure 3, bottom).

## Discussion

The key finding of our study was that in patients with septic shock, at the same level of MAP, dopexamine–norepinephrine enhanced GMBF more than epinephrine did.

With regard to systemic hemodynamics, epinephrine induced greater heart rate, cardiac output, oxygen delivery, and oxygen consumption than the combination of dopexamine and norepinephrine. These effects express the well-known strong β_1_-adrenergic stimulation induced by epinephrine [2] and the more balanced cardiac and vascular effects induced by the combination of dopexamine and norepinephrine. Epinephrine also induced a significant increase in arterial lactate, as has already been shown in patients with septic shock [9,11,12]. This effect may result from splanchnic hypoxia [12,13]. However, epinephrine could also increase arterial lactate independently of a defect of cellular oxygenation by stimulation of the skeletal muscle cell Na^+^, K^+^-ATPase, which accelerates aerobic glycolysis and consequently the production of lactate [14].

The effect of catecholamines on the hepatosplanchnic perfusion of septic patients remains controversial, depending on the method used to evaluate perfusion, the region studied (extended versus limited area) and the severity of patients (sepsis, severe sepsis, or septic shock). Indeed, dopexamine infusion during sepsis and septic shock increased splanchnic blood flow, but the fractional contribution of the regional blood flow to cardiac output decreased or remained unchanged [15,16]. In patients with septic shock, dopexamine alone or in combination with another catecholamine either did not change gastric mucosal pH [16,17] or increased it [18] but did not modify the gastric mucosal–arterial pCO_2 _gradient [17]. When using an original method to evaluate gastrointestinal mucosal perfusion (reflectance spectrophotometry), dopexamine infusion was shown to markedly improve the hemoglobin oxygen saturation of gastric mucosa [17]. However, these results were observed in uncontrolled studies. When patients with septic shock previously treated by norepinephrine were randomized to receive either dopexamine or dobutamine, the gastric mucosal–arterial pCO_2 _gradient was improved similarly in the two groups [19]. Similar conflicting results exist on the effects of epinephrine on intestinal perfusion in sepsis. Epinephrine infusion was found to decrease splanchnic blood flow, decrease gastric mucosal pH, and increase the gastric mucosal–arterial pCO_2 _gradient [15]. However, two studies found that GMBF, assessed as in our study by laser-Doppler flowmetry, increased during epinephrine infusion in patients with septic shock [8,9].

In the present study, both epinephrine and the combination of dopexamine and norepinephrine durably increased GMBF, but this effect was more pronounced with the combination of dopexamine and norepinephrine. The ratio between GMBF and cardiac output decreased during epinephrine infusion, whereas it did not change during dopexamine–norepinephrine infusion. Indeed, in comparison with dopexamine–norepinephrine, the increase in cardiac output allowed by epinephrine was not totally distributed to the gastric mucosa, as shown by a marked increase in estimated gastric mucosal resistance (MAP over GMBF ratio = +41% between T_0 _and T_1_). In the dopexamine–norepinephrine group, estimated gastric mucosal resistance increased only slightly (+9% between T_0 _and T_1_), supporting the hypothesis of a dopexamine-induced vasodilatation that counteracted the norepinephrine-induced vasoconstriction. These results are in agreement with those of experimental studies performed in septic rats demonstrating, by videomicroscopy, an improvement in intestinal mucosal blood flow during dopexamine infusion [20,21]. Our results must be interpreted in the light of the limitation of the laser-Doppler technique. Indeed, this technique does not take into account the heterogeneity of microvascular blood flow (a major characteristic of sepsis-induced microcirculatory disorders), because this technique measures the average velocity of all vessels comprised in the investigated volume [22,23]. Nevertheless, our results suggest that this technique is adapted to assess flow variations in the investigated territory under various pharmacological interventions.

The excessive production of reactive oxygen species during sepsis may be involved in cellular damage [24,25]. A recent study performed in critically ill patients showed a significant increase in oxidative stress, as assessed by plasma concentrations of thiobarbituric acid-reactant substances, both in patients with systemic inflammatory response syndrome and multiple organ failure and in non-survivors [26]. In rats, the reactive oxygen species generated by an intravenous administration of xanthine followed by xanthine oxidase induced a circulatory failure with a survival rate of 20% [5]. When the animals were pretreated by increasing doses of dopexamine, survival was enhanced to 70%. In our study, the production of malondialdehyde similarly increased in both groups and we did not find any influence of dopexamine on the production of reactive oxygen species.

## Conclusion

In septic shock, at doses that induced the same effect on MAP, dopexamine–norepinephrine enhanced GMBF more than epinephrine did. No difference was observed on oxidative stress. Our findings suggest that the combination of dopexamine and norepinephrine could be an interesting alternative in the treatment of the hemodynamic disturbances observed in septic shock.

## Key messages

• 	In septic shock, at doses that induced the same effect on mean arterial pressure, dopexamine–norepinephrine enhanced gastric mucosal blood flow more than epinephrine did.

• 	The combination of dopexamine and norepinephrine could be an interesting alternative in the treatment of the hemodynamic disturbances observed in septic shock.

## Abbreviations

GMBF = gastric mucosal blood flow; MAP = mean arterial pressure; SAPS II = Simplified Acute Physiology Score II; SOFA = Sequential Organ Failure Assessment

## Competing interests

The authors declare that they have no competing interests.

## Authors' contributions

PS conceived the study, participated in the design and execution of the study, the analysis of data and wrote the manuscript. BL participated in the execution of the study, the analysis of data and co-wrote the manuscript. PG and IM participated in the execution of the study and interpretation of the data. YM and EB supervised study planning, design, execution and analysis and co-wrote the manuscript. All authors read and approved the final manuscript.

## Figures and Tables

**Table 1:** General characteristics of study patients at inclusion

Parameter	Epinephrine (*n *= 10)	Dopexamine–norepinephrine (*n *= 12)	*p*
Age, years	67 ± 13	65 ± 10	0.797
Weight, kg	79 ± 15	77 ± 14	0.735
Height, cm	169 ± 9	168 ± 9	0.855
Sex ratio, M/F	8/2	9/3	1.00
SAPS II score	56 ± 17	52 ± 15	0.567
SOFA score	10 ± 4	10 ± 3	0.919
Fluid infusion^a^, ml	2,430 ± 980	2,521 ± 1,218	0.973
PaO_2_/FIO_2_, %	268 ± 103	191 ± 104	0.097
PEEP^b^, cmH_2_O	6	7 ± 2	

^a^This corresponds to the amount of fluid infused during the previous 24 hours; ^b^this applies to one and four patients in the epinephrine and dopexamine–norepinephrine groups, respectively.

Data are means ± SD or number of patients. FIO_2_, inspired oxygen fraction; PaO_2_, arterial pressure in oxygen; PEEP, positive end expiratory pressure; SAPS II, Simplified Acute Physiology Score II; SOFA, Sequential Organ Failure Assessment.

**Table 2:** Effects of epinephrine and dopexamine–norepinephrine on hemodynamics, oxygenation parameters and gastric mucosal blood flow

Parameter	Group	T_0_	T_1_	T_2_	T_3_	*p * (time effect)	*p * (treatment effect)	*p *(interaction)
MAP, mmHg	E	52 ± 7	81 ± 5	78 ± 4	87 ± 13	0.225	0.232	0.224
	D–N	56 ± 8	79 ± 8	80 ± 9	80 ± 13			
RAP, mmHg	E	11 ± 3	13 ± 3	12 ± 3	10 ± 3	0.028	0.597	0.987
	D–N	12 ± 3	13 ± 5	12 ± 3	10 ± 4			
MPAP, mmHg	E	23 ± 3	29 ± 6	29 ± 4	27 ± 5	0.289	0.141	0.453
	D–N	27 ± 6	31 ± 8	28 ± 6	27 ± 7			
PCWP, mmHg	E	14 ± 4	15 ± 3	15 ± 4	14 ± 3	0.201	0.268	0.342
	D–N	15 ± 4	16 ± 6	14 ± 4	12 ± 4			
HR, beats/min	E	94 ± 18	114 ± 24	113 ± 12	115 ± 14	0.991	0.023	0.699
	D–N	102 ± 17	108 ± 21	109 ± 19	109 ± 18			
SV, ml	E	68 ± 30	92 ± 39	87 ± 36	81 ± 33	0.032	0.531	0.334
	D–N	67 ± 21	81 ± 26	81 ± 18	78 ± 21			
CO, l/min	E	6.2 ± 2.4	10.1 ± 3.8	9.8 ± 4.1	9.2 ± 3.4	0.115	0.039	0.454
	D–N	6.8 ± 2.2	8.6 ± 2.7	8.8 ± 2.1	8.4 ± 2.6			
SVR, dyne.s/cm^5^	E	588 ± 188	635 ± 335	666 ± 400	803 ± 438	0.006	0.743	0.141
	D–N	621 ± 412	722 ± 417	672 ± 275	746 ± 353			
PVR, dyne.s/cm^5^	E	142 ± 84	122 ± 78	145 ± 103	143 ± 111	0.126	0.419	0.295
	D–N	198 ± 207	175 ± 166	128 ± 40	164 ± 77			
DO_2_, ml/min	E	624 ± 246	1,142 ± 442	1,160 ± 518	1,163 ± 415	0.500	0.009	0.918
	D–N	701 ± 220	967 ± 306	1,021 ± 275	968 ± 318			
VO_2_, ml/min	E	180 ± 46	275 ± 89	286 ± 53	250 ± 81	0.533	0.001	0.527
	D–N	204 ± 60	178 ± 78	206 ± 95	219 ± 108			
GMBF, pu	E	76 (61–107)	91 (62–136)	90 (72–133)	125 (90–160)	0.084	0.048	0.913
	D–N	106 (93–157)	137 (99–198)	133 (114–158)	165 (124–190)			
GMBF/CO, pu l^-1 ^min^-1^	E	18 (9–20)	11 (7–17)	15 (6–17)	15 (11–18)	0.128	0.015	0.686
	D–N	15 (10–20)	17 (11–25)	15 (10–23)	18 (11–39)			

Data are presented as means ± SD for normally distributed variables and as medians (25th to 75th centiles) for non-normally distributed variables. *p *values are those given by a two-way (time, treatment) analysis of covariance, the analysis being adjusted on baseline values (mixed model) for normally distributed variables or a non-parametric repeated-measures analysis, and also adjusted on baseline values (mixed model), performed on ranked data, for non-normally distributed variables. CO, cardiac output; D, dopexamine; DO_2_, oxygen delivery; E, epinephrine; GMBF, gastric mucosal blood flow; HR, heart rate; MAP, mean arterial pressure; MPAP, mean pulmonary arterial pressure; N, norepinephrine; PCWP, pulmonary capillary wedge pressure; pu, perfusion units; PVR, pulmonary vascular resistances; RAP, right atrial pressure; SV, stroke volume; SVR, systemic vascular resistances; VO_2_, oxygen consumption.
